# Strengthening data collection and use for quality improvement in primary care: the case of Costa Rica

**DOI:** 10.1093/heapol/czab043

**Published:** 2021-04-13

**Authors:** Madeline Pesec, Lauren Spigel, José María Molina Granados, Asaf Bitton, Lisa R Hirschhorn, Jorge Arturo Jiménez Brizuela, Michael Pignone, María del Rocío Sáenz, Dan Schwarz, Oscar Villegas del Carpio, Ira B Wilson, Eduardo Zamora Méndez, Hannah L Ratcliffe

**Affiliations:** Ariadne Labs, Brigham and Women’s Hospital & Harvard T.H. Chan School of Public Health, 401 Park Drive, Boston, MA 02215, USA; Division of General Medicine, Department of Internal Medicine, Brigham and Women’s Hospital, 75 Francis Street, Boston, MA 02115, USA; Ariadne Labs, Brigham and Women’s Hospital & Harvard T.H. Chan School of Public Health, 401 Park Drive, Boston, MA 02215, USA; Department of Quality Assurance, Costa Rican Social Security Administration, Second Avenue between 5th and 7th Street, San José, 10105, Costa Rica; Ariadne Labs, Brigham and Women’s Hospital & Harvard T.H. Chan School of Public Health, 401 Park Drive, Boston, MA 02215, USA; Division of General Medicine, Department of Internal Medicine, Brigham and Women’s Hospital, 75 Francis Street, Boston, MA 02115, USA; Center for Primary Care, Harvard Medical School, 25 Shattuck Street, Boston, MA 02115, USA; Department of Medical Social Sciences, Northwestern University Feinberg School of Medicine, 420 E Superior Street, Chicago, IL 60611, USA; Costa Rican Social Security Administration, Second Avenue between 5th and 7th Street, San José, 10105, Costa Rica; Department of Internal Medicine, Dell Medical School University of Texas, 1501 Red River Street, Austin, TX 78712, USA; School of Public Health, University of Costa Rica, Calle la Cruz 26, San José, Mercedes, Costa Rica; Ariadne Labs, Brigham and Women’s Hospital & Harvard T.H. Chan School of Public Health, 401 Park Drive, Boston, MA 02215, USA; Division of Global Health Equity, Department of Medicine, Brigham and Women’s Hospital, 75 Francis Street, Boston, MA 02115, USA; Department of Medicine, Harvard Medical School, 25 Shattuck Street, Boston, MA 02115, USA; Health Service Delivery Strengthening Department, Costa Rican Social Security Administration, Second Avenue between 5th and 7th Street, San José, 10105, Costa Rica; Department of Health Services, Policy & Practice, Brown University School of Public Health, 121 South Main Street, Providence, RI 02903, USA; Costa Rican Social Security Administration, Second Avenue between 5th and 7th Street, San José, 10105, Costa Rica; Ariadne Labs, Brigham and Women’s Hospital & Harvard T.H. Chan School of Public Health, 401 Park Drive, Boston, MA 02215, USA

**Keywords:** Primary care, primary healthcare, clinical, health information system, Central America, Costa Rica

## Abstract

Costa Rica is a bright spot of primary healthcare (PHC) performance, providing first-contact accessibility and continuous, comprehensive, coordinated, and patient-centered care to its citizens. Previous research hypothesized that strong data collection and use for quality improvement are central to Costa Rica’s success. Using qualitative data from 40 interviews with stakeholders across the Costa Rican healthcare system, this paper maps the various data streams at the PHC level and delineates how these data are used to make decisions around insuring and improving the quality of PHC delivery. We describe four main types of PHC data: individual patient data, population health data, national healthcare delivery data, and local supplementary healthcare delivery data. In particular, we find that the Healthcare Delivery Performance Index—a ranking of the nation’s 106 Health Areas using 15 quality indicators—is utilized by Health Area Directors to create quality improvement initiatives, ranging from education and coaching to optimization of care delivery and coordination. By ranking Health Areas, the Index harnesses providers’ intrinsic motivation to stimulate improvement without financial incentives. We detail how a strong culture of valuing data as a tool for improving population health and robust training for personnel have enabled effective data collection and use. However, we also find that the country’s complex data systems create unnecessary duplication and can inhibit efficient data use. Costa Rica’s experience with data collection, analysis, and use for quality improvement hold important lessons for PHC in other public sector systems.

**Key messages**

- Costa Rica’s robust primary healthcare (PHC) system is buoyed by a vast data system, made up of many different data streams.
- The Health Care Performance Index, one of these data streams, ranks the country’s 105 Health Areas on the quality of healthcare they provide and stimulates quality improvement initiatives.
- The PHC system has a strong culture of valuing data and has invested in the training necessary for data to effectively drive decision-making.
- Overly complex data collection and storage systems currently hinder efficiency, but new digitalized health records may alleviate bureaucratic redundancy.

## Introduction

Primary healthcare (PHC) is the cornerstone of strong healthcare systems, and strengthening PHC will be essential to achieving Universal Health Coverage, as outlined in the Sustainable Development Goals (Macinko *et al.*, 2009; Pettigrew *et al.*, 2015). In 2018, the global health community united around the Astana Declaration for PHC, which reaffirmed the world’s commitment to PHC as the basis of strong health systems as first put forth in the Alma Ata Declaration of 1978 (Declaration of Astana, 2018). However, PHC is often under-funded and can be of low quality, particularly in low- and middle-income countries (Das and Hammer, 2014). Efforts to improve PHC are complicated by the fact that many countries lack robust data about PHC performance (Veillard *et al.*, 2017). Therefore, as policymakers work to strengthen PHC, careful focus on improving the collection and use of data to drive accountability and quality improvement is needed. In this context, an analysis of the experience of Costa Rica, a middle-income country that is successfully collecting and using data for PHC improvement, can be instructive.

**Figure 1:**
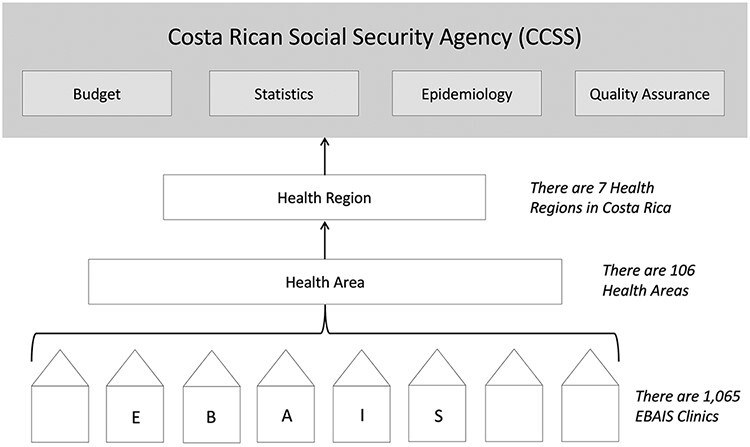
Primary Health Care System Organization.

In 1994, Costa Rica underwent a major PHC reform (Spigel *et al.*, 2020), creating a system that provides a comprehensive, coordinated, continuous, patient-centred first point of contact for its citizens (Pesec *et al.*, 2017a; Bitton *et al.*, 2018). In 2019, Costa Rica’s life expectancy was second only to Canada in the Western Hemisphere, and the country performs in the top 10% of low- and middle-income countries on effective PHC coverage and primary care–related health outcomes (Pesec *et al.*, 2017b; United Nations Development Programme, 2019). In the 1994 reform, the responsibility for all public sector healthcare delivery (including public health efforts) was consolidated under the Social Security Administration [Caja Costarricense de Seguro Social (CCSS)]. PHC delivery is organized into seven Health Regions, 106 Health Areas and 1065 primary care clinics, known as Equipos Básicos de Atención Integral de Salud (EBAIS) (Comprehensive Basic Primary Healthcare Teams). Figure 1 describes the organization of PHC. Multidisciplinary EBAIS teams consist of a doctor, a medical assistant, a community health worker [known as Asistente Técnico de Atención Primaria (ATAPs)] and a medical data clerk [known as Registros de Salud Clerk (REDES)]; each team cares for a geographically empanelled population of $\sim$4000 individuals. These teams work collaboratively to deliver multidisciplinary, preventive and curative care to all Costa Ricans.

Previous work by Pesec *et al*. identified four critical components of healthcare reforms undertaken in the 1990s that contributed to this success (Pesec *et al.*, 2017b). One component—strong measurement and feedback loops for PHC—was hypothesized to be essential to the success of the other three and the long-term sustainability of Costa Rica’s reform efforts. However, little information about Costa Rica’s system for data collection and utilization exists in the international literature. The goal of this paper is to identify the sources of PHC data in Costa Rica’s healthcare system and describe how these data are used for quality improvement. We had three central questions: (1) What are the main sources of PHC data in Costa Rica? (2) Which sources of data have been most important for stimulating quality improvement? and (3) What enablers and barriers to effective data collection and use can be identified?

## Methods

The results presented here were part of a larger study to document the history, implementation and challenges of PHC reform in Costa Rica. This study used a qualitative methodology and conducted in-depth, in-person, and semi-structured interviews with 40 key informants from different levels of the Costa Rican health system (Table 1) (Tong *et al.*, 2007). Using convenience sampling based on availability, and supplemented with snowball sampling, we aimed to capture diverse perspectives from key informants from all levels of the healthcare system with experience as producers and consumers of PHC data. Given the diversity of topics discussed as part of the larger study, we did not attempt to reach saturation. One author (MP) conducted over 100 hours of interviews exploring the use of data in PHC, with the average interview lasting 72 minutes. Initial data collection took place in 2017 and was supplemented in 2019 with four additional interviews for clarification of emerging themes.

**Table 1.** Positions of key informants

Key informants by position	*n*
Costa Rica Social Security Administration	13
Quality Assurance Department	6
Health Network Department	3
Health Service Delivery Strengthening	1
Budget Department	2
Statistics Department	1
Health Region	4
Health Area	18
Health Area Directors	6
Health Area Staff	6
Primary Care Providers	3
Medical Data Clerk	3
Costa Rican Academics	5
Total	40
Key informants by training	*n*
Medical Doctor	18
Administrator	22
Total	**40**
Key informants by gender	*n*
Male	26
Female	14
Total	40

An interview guide directed conversation with key informants. The interview guide was designed to identify details about data streams of relevance to PHC and was developed based on two frameworks. The first was the Data to Improvement Pathway, which describes a six-step process for using raw data to drive improvements (Joint Learning Network for Universal Health Coverage, 2018). The second was the Adaptive Management framework, which describes an interactive feedback loop for developing quality improvement projects based on data (Williams and Brown, 2014).

Interviews were conducted, recorded and transcribed in Spanish. Two authors (LS and MP) coded transcriptions in Dedoose© software. A codebook was developed through inductive and deductive methods. Initial codes were developed based on the Data to Improvement Pathway and the Adaptive Management Framework to answer our research questions. Additional codes were added inductively as new themes and data streams emerged. Fifteen percent of interviews were double coded by LS and MP until consistency was reached between coders. Thereafter, MP and LS coded individually, meeting regularly to review codes and reach consensus. Authors HLR and LRH resolved discrepancies.

Additionally, we reviewed documents provided by our key informants as well as publicly available documents published by the CCSS and the Quality Assurance Department to supplement information obtained from interviews. The author’s institute IRB determined that this work was not human subject research and therefore did not need formal IRB review. All informants gave verbal consent, were informed that the interview was optional and were advised they could stop the interview at any time.

## Results

### What are the main sources of PHC data in Costa Rica?

**Figure 2:**
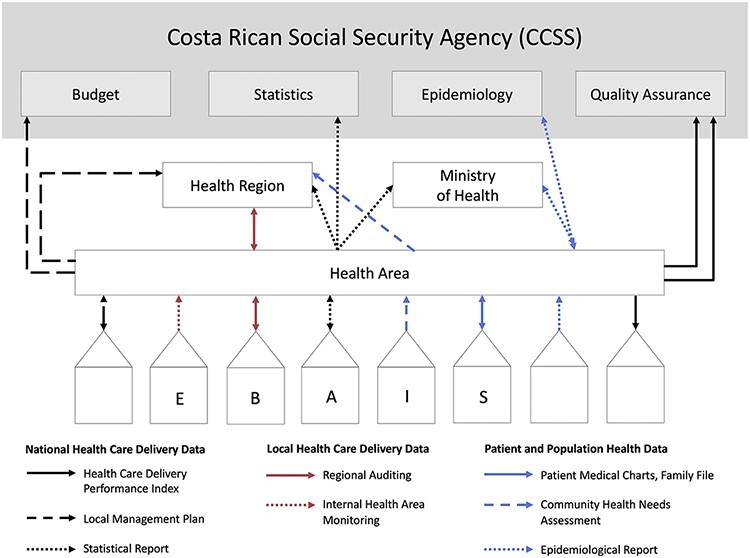
Data flow at the primary care level.

We identified nine major PHC data streams in Costa Rica (Table 2), which can be grouped into four categories: data on individual patients, population health data, national healthcare delivery data, and local supplementary healthcare delivery data. Figure 2 illustrates the collection and feedback of these data streams from local to national levels.

**Table 2.** Data in Costa Rica’s primary healthcare system

	Description	Frequency at which data is collected	Who collects data?	Where is data sent?	Are there targets for each metric?	Is there feedback of data to Health Areas?	How is data used at a local level?	How is data used at a national level?
Individual patient health data								
Patient Medical Records	Patient medical charts, completed by EBAIS nurses and physicians	Daily	Physicians and nurses	Stored at the Health Area	No	No	Used for clinical care	N/A
Family File	Charts documenting the social determinants of health, completed in the field by community health workers	Daily	Community health workers, known as ATAPs	Collected at the Health Area	No	No	Used to identify patients in need of extra healthcare/support services and to direct educational activities	N/A
Population Health Data								
Community Health Needs Assessment	An assessment of the causes of morbidity mortality and unmet healthcare needs	Once every 2 years	Health Area administrators and medical team with input from the community.	Regional and national levels	No	No	By design should be used to guide the programming and to identify areas of need	N/A
Epidemiological Report	Documentation of reportable diseases, mainly infectious diseases, with the aim of preventing spread	Weekly	REDES and the epidemiological team at each Health Area.	Epi departments at the regional and national levels, as well as to the Ministry of Health.	No	Yes, for epidemiological surveillance.	Can stimulate Health Areas to conduct epidemiological sweeps of certain neighbourhoods and can mobilize additional resources for infection control	To monitor and track emerging threats by CCSS and MoH. Used to mobilize resources during outbreaks
National healthcare delivery data								
Local Management Plan	Data repository with over 300 indicators used for long-term strategic planning of the Health Area	Four times per year	Data compiled by the REDES, and input into the computer system by administrators.	To the Health Region and to the national budget department	Yes, based on historical performance and negotiation between HA, HR, and budget department	Yes, with the region. Health Area’s progress against targets is discussed.	Data used to monitor production. Can stimulate changes in programming to achieve targets	Budget Department tracks if Health Areas are using their budgets appropriately to conduct activities
Statistical Report	Catalogue of all diagnoses seen, procedures performed and appointments by the Health Area	Monthly	REDES	To the region, and to the national statistical area.	No	No	Used to make sure that all departments are seeing the appropriate number of patients each month. Not used to stimulate quality improvement activities	CCSS departments can petition statistical department for access to their records, but this is difficult
The Healthcare Delivery Index	Ranking of the quality of all Health Areas, based on direct evaluation of patient charts	Annually	Investigators from the Quality Assurance Department collect directly from patient charts.	Sent to regional and national management. Published publicly.	Yes	Yes, with advice for improvement on these targets if Health Area desires.	Used extensively at the local level to stimulate quality improvement activities in order to perform better next year on the Index	Used to ensure the quality of care provided at the local level
Local supplementary healthcare delivery data								
Regional Auditing	Tracking of general and region-specific targets for Health Areas	Annually	REDES at the Health Area collate	To the region	Yes, region specific	Variable	Used at regional level to ensure quality of Health Areas	N/A
Internal Health Area Monitoring	Flexible, supplemental data at the local level	Variable (monthly-tri-yearly)	REDES at the Health Area collate	Nowhere, kept at Health Area	Variable	Variable	Variable—quality improvement and monitoring	N/A

#### Individual patient data

There are two main sources of data on individual patients. The first is standard patient medical charts, accessible by the patient’s providers. The second is the Family File (*Ficha Familiar)*, filled out by community health workers (ATAPs) in EBAIS teams during annual home visits. It contains information about citizens’ social and health situations, anthropometric measurements and vital signs. It is also used by the Health Area to understand social determinates of health and to risk stratify, identifying those in need of more frequent visits. Patient charts and the Family Files are now electronic, but during data collection in 2017, patient charts were largely on paper.

*Can you imagine the power that these data have? You have information about all the people in the household, the house itself, the community, and then [from medical charts] who hasn’t shown up for treatment. For example, we have identified 100 pregnant women through the Family File but only 80 have come to the clinic. Why haven’t they come? Now we can develop strategies to engage them* (Scholar, Department of Public Health, University of Costa Rica).

#### Population health data

Two main sources of data measure the health of populations. The first is the weekly epidemiological report compiled by medical data clerks (REDES) for all mandatory reportable diseases. Examples of reportable diseases include infant mortality, diarrhoea, influenza and dengue. This report is sent to the epidemiology department at the Health Region, national CCSS, and the Ministry of Health for disease tracking and follow-up as needed. Epidemiological teams at the Health Area also use the report to identify source cases and quarantine individuals as necessary, in coordination with the CCSS and Ministry of Health.

The second data source, the Community Health Needs Assessment, is conducted by each Health Area once every 2 years and maps the principal causes of morbidity and mortality in the Health Area. Data come from the Family File in addition to data provided by other community organizations and needs assessments are created collaboratively with the community, with forums and focus groups providing feedback on early drafts. This assessment is then used to create programming that meets the needs of the population.

#### National healthcare delivery data

The CCSS mandates data collection on PHC delivery through three national programmes: the Statistics Report, the Local Management Plan, and the Healthcare Delivery Performance Index.

The Statistics Report is a monthly document of diagnoses seen, procedures performed, and appointments completed by the Health Area and its EBAIS clinics; it represents the major source of information for the CCSS about its internal production. Data are collected by medical data clerks (REDES) and summary reports are produced. Reports are sent to the Health Region and CCSS Statistics Department, which are responsible for data collation across Health Areas. Health Area Directors and Health Region Directors turn to the statistics report for up-to-date information on the production of the primary care clinics (EBAIS) and the Health Area.

*Based on this report month by month… [Health Area Directors] use this information* *…* *to see what I can improve. Where do I have to change strategies? Where do I have to improve production?* (Health Region Director).

The Local Management Plan is a yearly document with over 300 indicators measuring processes of healthcare delivery at the Health Area. Nearly every activity conducted in the Health Area is recorded here, making its scope much larger than the Statistics Report. This database is used for long-term planning rather than day-to-day quality improvement activities. The national Budget Department of the CCSS uses it to ensure Health Areas carry out planned activities and to design the next year’s budget. Health Areas also use the document to track progress toward local targets. For example, if the Health Area aims to complete 5000 Pap smears per year, and they are not on track to achieve this, they can increase the number of gynaecologic visits to achieve their target.

The Healthcare Delivery Performance Index (hereafter referred to as ‘the Index’) is a yearly evaluation by the Quality Assurance Department within the CCSS that ranks the coverage and quality of PHC provided at the 106 Health Areas from best to worst. The Index is constructed by directly reviewing a sample of patient charts to assess performance on 15 indicators. Based on the Index results, Health Areas then create quality improvement activities to improve their performance.

#### Local supplementary healthcare delivery data

There is considerable flexibility in the system for Health Regions and Health Areas to set their own priorities and create additional data collection systems that meet their specific needs. Two examples of this are regional auditing of the Health Area and the Health Area’s own internal monitoring.

Regional auditing of Health Areas enables tracking of region-specific targets. Health Regions are required to audit and monitor their Health Areas, but the means by which they do so is not strictly specified, leading to a variety of approaches both in indicator selection and data use. The Health Region also compiles data for additional ad hoc requests for information from other CCSS departments.

In addition, all respondents with insight into Health Areas reported supplemental data collection, analysis, and monitoring activities to track areas of interest, such as mental health visits or urgent care utilization. Health Area Directors require a dynamic set of information to manage their clinic on a day-to-day basis and answer specific questions, and they have adopted a diverse means of collecting this information.

*Management is very broad and very dynamic. So tomorrow I’ll need some type of information that maybe in this moment today I do not need… Even with all this information I feel a little bit blind sometimes. I am not sure how others [who do not have access to data] do it* (Health Area Director).

### Which sources of data have been most important for stimulating quality improvement activities?

While all of the previously described data streams contribute to the landscape of data at the PHC level, our informants identified the Index as one of the primary mechanisms for driving accountability for quality improvement in PHC. The sources of data used to create the Index are highlighted by the solid black in Figure 2. This section describes in detail the process used to create the Index, how the Index is reported to spur quality improvement activities, and changes in outcomes driven by the Index.

#### Creation of the Index by the Quality Assurance Department

The Index is calculated annually by the national CCSS Quality Assurance Department with the aim of measuring and ranking the quality of healthcare delivered at each Health Area. The Index consists of 15 indicators (Table 3) that have been internationally validated are associated with improved patient outcomes, and can be extracted from patient charts. Each indicator has a national goal set by the CCSS that Health Areas should aim to achieve, and these goals are incrementally increased each year. In recent years, there has been an effort to measure intermediate health outcomes rather than healthcare processes. For example, instead of measuring the number of diabetic foot exams, the Index now measures the percentage of patients who achieve glycaemic control.

**Table 3.** Indicators used in the Healthcare Performance Delivery Index

Adult health indicators	Maternal health indicators	Child health indicators
Percent adults with hypertension who achieved blood pressure control	Percent of pregnant women seen for prenatal visit before 13 weeks’ gestation	Percent of children under 1 year of age who received basic vaccinations
Percent adults with type 2 diabetes with LDL control	Percent of pregnant women with an HIV test before 20 weeks’ gestation	Percent of children aged 1–2 years with complete vaccinations
Percent adults with type 2 diabetes with blood pressure control	Percent of pregnant women with a syphilis test before 20 weeks’ gestation	Percent of children aged 6 months to 2 years who had a haemoglobin test
Percent adults with type 2 diabetes with HbA1c control	Percent of post-partum women seen before 8 days post-partum	Percent of anaemic children from 6 months to 2 years fully treated
Percent of women aged 35–65 with a pap smear in the last 2 years		Percent of newborns seen before 8 days of life, early in the post-natal period
Percent elderly who received complete vaccinations

*The process indicators, they measure things close to what is important. For example, I could measure if you woke up at the right time, if you showered quickly, if you ate your breakfast.. We could evaluate this whole process. But really what I care about is the outcome* *—* *whether you are here with me or not*. (Quality Assurance Department Evaluator).

Indicators are selected and approved annually by all members of the Quality Assurance Department and are refined over time based on changing burden of disease, emerging quality measurement science, and the types of information available in patient charts. Beginning in 2014, the importance of maintaining continuity in indicators to allow comparison over time was identified and, since then, the Quality Assurance Department has aimed to strike a balance between keeping indicators current, while also allowing for comparison from year to year.

**Figure 3:**
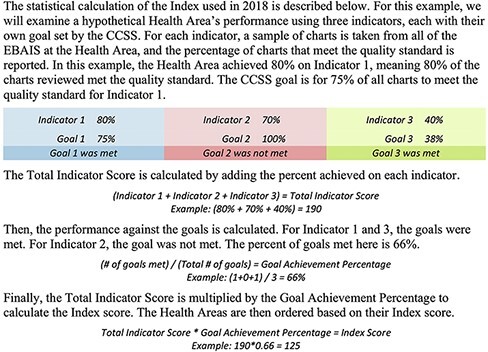
Statistical calculation of the Index.

To calculate the Index, investigators from the Quality Assurance Department directly review patient charts by selecting a representative sample (40–90 patient records) per indicator per Health Area, chosen at random from the EBAIS clinics in the Health Area. In 2018, the Quality Assurance Department evaluated 43 372 patient charts (Direccion Compra de Servicios de Salud, 2019). To create the Index, the Quality Assurance Department factors in both the performance on each indicator and the number of indicators that met the national goal, as shown in Figure 3. No scoring adjustments are made based on Health Area resources, population size, or population risk profile. This was an intentional decision based on the philosophy that all Costa Ricans deserve the same quality of care, no matter where they live. Index results are published publicly on an annual basis and are also fed back to the Health Regions and Health Areas through formal meetings.

#### Quality improvement spurred by the Index

If the Health Area scores in the bottom 20% of Health Areas on the Index, they are required to submit a remediation plan to the Health Region outlining their proposed strategies for improvement. Regardless of their Index ranking, all Health Areas are encouraged to review their results and make improvements for the coming year. Health Regions hold meetings where Health Areas can discuss their strategies for improvement in order to share successful strategies between Health Areas.

**Figure 4:**
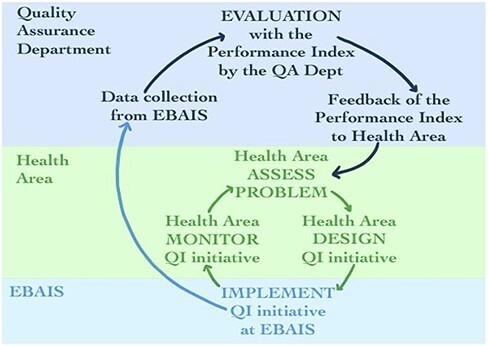
The Index stimulates quality improvement.

Based on the adaptive management framework developed by Williams and Brown, we mapped how the Index is used to simulate quality improvement, illustrated in Figure 4. Although exact quality improvement activities vary by Health Area, the core activities of adaptive management are generally consistent. After the Health Areas receive feedback of the results of the Index from the Quality Assurance Department, they internally review the results, sometimes working with the Quality Assurance Department to unpack their scores and glean additional information about their performance.

*After one Health Area Director declined heavily in the Index, she remarked, ‘* *well, I will have to give explanations, but the most important thing is that I start working. And that I start working since the first hint that the results would be bad*.’ (Health Area Director).

Health Areas then begin to identify areas for improvement, looking for identifiable patterns in the data that may suggest issues such as confusion among doctors in the application of clinical guidelines, poor performance centralized in specific EBAIS, or difficulties with laboratory testing. The Health Area may review some patient charts internally and interview clinicians to see if they can elucidate areas for improvement.

*The point of the Index is to stimulate the Health Area to look internally and figure out where they can improve.* (Quality Assurance Department Evaluator).

Then, the Health Area will design and implement a quality improvement initiative to improve their performance. There is heterogeneity in the design process, since each Health Area Director is encouraged to craft interventions that they believe will address their Health Area’s needs. There are limitations to the interventions that the Health Area can design, as they often lack extra resources to spend, and significant changes such as changing hours of operation or adding laboratory capacity must be approved by the upper level of the CCSS, which can take time. Many different Health Area Directors described similar strategies, centred around education on why the indicator is important for patient health and coaching on how best to provide care in a particular context to achieve the target. Generally, this is a collaborative, iterative process between the Health Area management and the clinicians that is founded on the assumption that every clinician wants to do his/her best, they just need the tools and strategies to achieve their goals. If these collaborative strategies fail, formal sanctions or even a firing process may be pursued; however, this is a last resort, difficult in public institutions, and reportedly happens infrequently. Certainly, not all interventions follow the above format, but this was commonly described across the Health Areas interviewed.

*I think that everyone should be convinced of what they do and why they do it… my objective is not to achieve the goal [for each indicator]. My objective is the ‘* *why’* *behind this goal. I must be sure that I am doing my daily work well, and trust that this will allow me to achieve my goal*. (Health Area Staff and former Primary Care Physician).

Many Health Areas also conduct routine internal sampling on all the Index’s indicators, regardless of specific quality improvement projects, to ensure that they are performing well throughout the year. However, there is heterogeneity in the manner and frequency of the internal samplings. Multiple directors noted that when they decreased the frequency of routine internal sampling, their performance on the Index fell. Continual internal monitoring was described as a key to success on the Index.

Informants reported that Health Areas care deeply about their performance on the Index and go through cycles of improvement to perform better the next year, even if not formally required. Notably, this engagement occurs without any financial incentives. Health Area providers cited two main reasons for this widespread commitment to and engagement with the Index. First, they believe that their performance on the Index closely mirrors the quality of care that patients experience and feel they have a professional commitment to provide high quality care. They trust the Index is appropriate, reliable and valid. Second, since the results of the Index are published publicly, they have a desire to perform well on the Index to protect their own professional reputation and personal pride. Health Area Directors who fail to work to improve their performance on the Index consistently over many years may have their job performance called into question.

#### Outcomes of the Index

Many positive outcomes of the Index were identified by our key informants. First, the Index is particularly influential in fostering a culture of using data to drive decision-making. The Index is a core part of a virtuous cycle in which data are used to drive decision-making, spurring improvement, and thus demonstrating the value of data use. This reinforces a culture that prioritizes data-driven decision-making, leading to even further improvement.

*If you are a doctor and you get a bad grade [on the Index] this motivates you to improve. If I tell you that you are not treating all your patients optimally, then what do you think? You are the doctor of this town. You have to figure out what you have to do to improve.* (Primary Care Provider).

Informants reported that the Index supports a sustained emphasis on quality indicators and helps Health Areas stay committed to their quality improvement efforts. Many Health Areas that have performed poorly on the Index subsequently engage deeply with quality improvement efforts and have a commensurate increase in Index performance. The Index also helps Health Areas that are doing well to guard against complacency; if their performance falls on the Index, they are spurred to re-engage with their improvement strategies. One informant posited that this cyclic engagement with the Index demonstrates exactly why the Index is critical for PHC—that without the Index, Health Areas might become lax with their efforts and quality could potentially fall.

*When [one Health Area] dropped in the ranking from first to 40th the director there nearly killed himself. Why did the Health Area fall? Well, they relaxed, they dedicated themselves to other things and they didn’t pay attention to the Index indicators. But, the next year, they worked very hard and were back on top*. (Scholar, Department of Public Health, University of Costa Rica).

**Figure 5:**
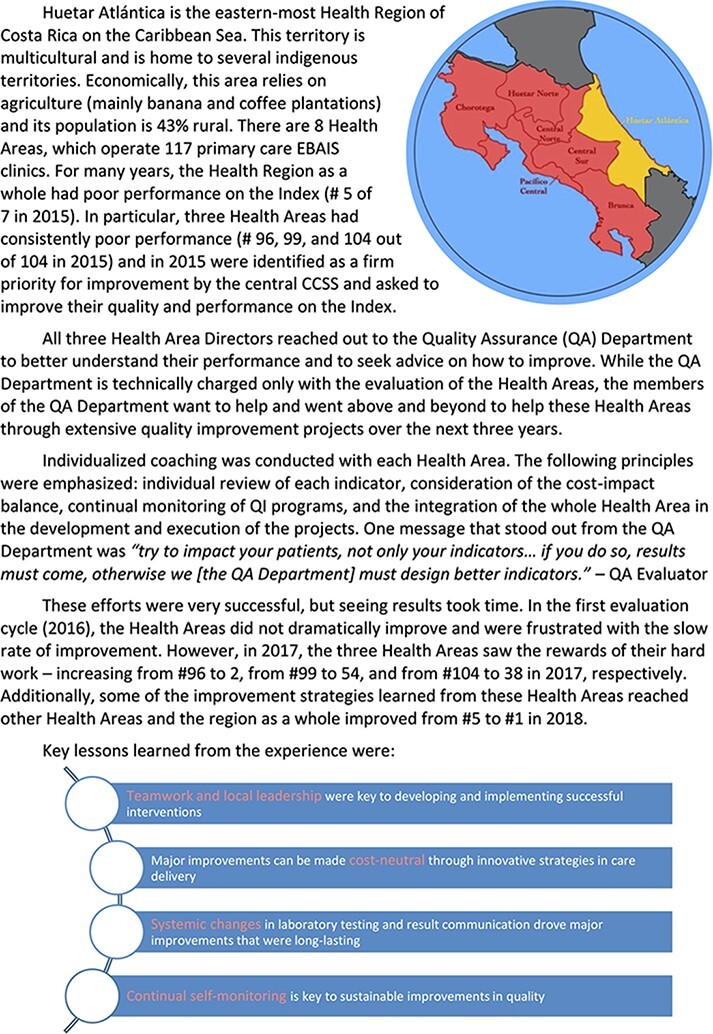
Huetar Atlántica: A Story of Quality Improvement.

Second, based on the Index and collaboration with the Quality Assurance Department, many Health Areas have been able to make improvements in quality. Over the past four years, the Quality Assurance Department has tracked performance on its indicators across the country and has noted sustained, incremental improvement across many different categories over time (enumerated in Table 4), without any increase in resources or funding. Figure 5 illustrates the improvement process in one Health Region, Huetar Atlántica. Through close collaboration with the Quality Assurance Department evaluators, this Health Region was able to improve from the fifth ranked Health Region of seven, to the first. Through intensive individualized coaching and through systematic changes to laboratory result delivery, Huetar Atlántica improved its performance on the Index and the quality of care it delivered to its population.

**Table 4.** Change in Index Indicators, 2014–2018

Indicator	2014	2018	% change
Adult Health Indicators			
Percent adults with hypertension who achieved blood pressure control	63.2 (In 2016)	65.7	4% Increase
Percent adults with type 2 diabetes with LDL control	34	40	18% Increase
Percent adults with type 2 diabetes with blood pressure control	50	55	10% Increase
Percent adults with type 2 diabetes with HbA1c control	39	44	13% Increase
Percent of women aged 35–65 with a pap smear in the last 2 years	35	37	6% Increase
Percent elderly who received complete vaccinations	61%	68%	11% Increase
Maternal Health Indicators			
Percent of pregnant women seen for prenatal visit before 13 weeks’ gestation	80	79	1% Decrease
Percent of pregnant women with an HIV test before 20 weeks’ gestation	70	76	9% Increase
Percent of pregnant women with a syphilis test before 20 weeks’ gestation	74	78	5% Increase
Percent of post-partum women seen before 8 days post-partum	83	85	2% Increase
Child health indicators			
Percent of children under 1 year of age who received basic vaccinations	92	94	2% Increase
Reached a maximum of 98% in 2017			
Percent of children aged 1–2 years with complete vaccinations	95	96	1% Increase
Reached a maximum of 98% in 2017			
Percent of children aged 6 months to 2 years who had a haemoglobin test	67	76	13% Increase
Percent of anaemic children from 6 months to 2 years fully treated	26	66	154% Increase
Percent of newborns seen before 8 days of life, early in the post-natal period	78	81	4% Increase
Additional indicators			
Percent of pregnant women who were seen at an EBAIS clinic	83	87	5% Increase
Percent of post-partum women who were seen at an EBAIS clinic	71	76	7% Increase
Percent of total population predicted to have hypertension diagnosed with hypertension	38	42	10% Increase
Percent of total population predicted to have diabetes diagnosed with diabetes	41	46	12% Increase

Data source: (Direccion Compra de Servicios de Salud, 2019).

Overall most Index indicators (with the exception of the percent of pregnant women who were seen for a prenatal visit before 13 weeks’ gestation) improved over the time period of 2014–2018.

Despite the many positive outcomes of the Index, informants identified some negative, unintended consequences. The focus on the Index indicators may leave less time for physicians to interact with patients and less time for Health Area Directors to explore priorities and health innovations that are not captured in the Index, but which may be beneficial to their population. For example, after a poor performance on the Index, one Health Area Director interviewed had to put an initiative to create a multidisciplinary psychiatric clinic on hold so that the Health Area could focus on improving its Index performance for the next year. Health Area Directors also noted that it was easier to complete short-term interventions to increase their performance on the Index rather than invest in large, structural changes. For example, in order to increase the rates of Pap smear screening, many Health Areas conduct screening campaigns, which increases screening rates in the short term but may not lead to a sustainable increase in Pap smear rates in the future.

*Obviously the systemic strategies are better because they last longer, but there are still some instances where it is reasonable to do a campaign. For example, in our rural areas there are many women who do not want their Pap smear done by a man and so once every 6 months we send a female doctor out and that works well for that population. But even that won’t last forever*. (Health Area Director).

### What enablers and barriers to effective data collection and use can be identified?

Two main enabling factors for the overall system of data collection and use emerged from discussions with key informants. The first is a strong culture of valuing data as a tool to drive improvements in patient and population health. As has been previously described, a defining characteristic of the Costa Rican health system is its commitment to quality and equity (Pesec *et al.*, 2017b). Key informants described a sense of accountability for achieving positive health outcomes for individuals and populations, and our results indicate that data collection and use has been internalized as a key tool for meeting this responsibility. This has been intentionally cultivated over time—antecedents of the Community Health Needs Assessment and the Family File have existed in some areas of Costa Rica since the 1970s, and the 1990s reforms reinforced data as critical to the success of PHC as a valuable tool to improve the health of Costa Ricans.

*When they train ATAPs (community health workers) and REDES (medical data clerks) in data collection, they tell them, ‘this information is important for you as a healthcare professional, not because it is important to send to the central level, but because you know that you need this information about as much of your population as possible*. (Scholar, Department of Public Health, University of Costa Rica).

Many informants described a deep commitment to the accurate collection and preservation of data. Among many informants, there was a consensus that data are essential insofar as they provide guidance for the Health Area and have a direct impact on patient health.

*To just have data stored in a closet doesn’t make sense. The idea is for the data to have utility that can be helpful for decision-making at the local level… all the decisions I make are based on the data I have available to me*. (Health Area Director).

As described above, this can be a virtuous cycle, wherein data are used to drive improvement, demonstrating the value of data and reinforcing a culture that values data as a tool for improving health.

The second enabling factor is the high level of technical and managerial proficiency that supports effective data collection, analysis, and use. This proficiency has been intentionally cultivated over the past 25 years through uptraining of staff—including each member of the EBAIS team—in data science. For example, medical data clerks (REDES) and community health workers (ATAPs) are trained extensively in data collection and collation. Primary care physicians and nurses have training in epidemiological principles and quality improvement, so they feel confident making decisions based on data. Additionally, in order to create the Index, the Quality Assurance Department investigators undergo extensive training in statistics and epidemiology. To complement this technical proficiency, Health Area Directors and Administrators receive specific training in Health Area management in addition to their training in epidemiology and data analysis, enabling them to effectively generate change based on their data.

The main barrier to data collection and use identified by key informants was inefficiency, including duplicative reporting and inconsistent feedback. While the ability of Health Regions and Health Areas to institute their own data collection and monitoring processes allows them flexibility, at the same time, the sheer quantity of data collected was described by informants as overwhelming and may act as a barrier to efficient data use. For example, solely for monitoring the quality of healthcare provided to its population, the Health Area must juggle, at the minimum, over 300 indicators from the Local Management Plan, 15 indicators from the Index, over 25 pages of indicators for the statistical reports, dozens of indicators for the Health Region, and any internal indicators they use to check their progress.

Informants at the Health Area level described an onslaught of data that makes it difficult for them to complete their other job activities because they feel they are always reporting or collating data. Informants expressed a desire for a simplified data system that would eliminate the redundancies in the current system and expressed hope that the new electronic medical record would assist in this process.

*This is one of the weaknesses that we have always identified in our [information] system: that in all of our data instruments, [there is] disarticulation, because the institution has not made good use of our data. Each different department asks for different information with this [data] instrument or that instrument, there is not a coherent vision.. we have to consolidate this information*. (Health Region Staff).

## Discussion

In this article, we describe how data systems in Costa Rica support high-quality PHC. We identified nine major data streams measuring individual and population level health, as well as the quality of healthcare delivery at the local and national levels. We also found that there is still flexibility for local levels to implement additional data collection measures, which reportedly empowers Health Areas to monitor and improve their healthcare quality but can also create data duplication. National, regional, and local data streams often operate independently from one another, and Health Areas are responsible for managing large amounts of data and sending appropriate reports weekly, monthly, and yearly.

Of the nine data streams identified, the Healthcare Delivery Performance Index was identified as particularly influential for service delivery quality improvement at the Health Area level. A scientifically rigorous, independent analysis that compares Health Areas against one another, the Index is valued and has led to sustained, incremental improvement across a variety of healthcare quality indicators over the past 25 years.

Globally, there is a growing recognition of the critical role that data use for quality improvement must play to improve PHC, and Costa Rica is not alone in endeavouring to effectively measure and improve PHC (Joint Learning Network for Universal Health Coverage, 2018; Ohkubo *et al.*, 2013). For example, Chile, a country which has also made investments in PHC over the past decade, measures and incentivizes quality at its PHC centres through two systems The first, known as ‘Health Goals’ is a pay-for-performance scheme for front-line PHC providers, where high performance on 10 indicators can earn providers a 16% salary bonus (Ahumada *et al.*, 2016). The second, called PHC Activity Indicators, are a set of 16 indicators measuring PHC in each municipality; performance on these indicators determines the municipalities’ capitation payments. Argentina has also employed a results-based financing (RBF) programme that allocates funds based on the PHC coverage achieved by each province (Silva *et al.*, 2016). Primary care coverage is defined as the percentage of eligible children, adolescents and adults who have received at least one high priority health services in the last year. Argentina couples these financial incentives with peer recognition and working environment improvements to boost provider motivation.

What sets Costa Rica apart from many other low- and middle-income countries is that it has created an effective way to drive sustained improvement in healthcare quality by ranking their Health Areas through the Index without attaching financial incentives to high performance and without risk-stratification for socio-economic status or available resources. While public feedback and structured improvement plans are important components of the Index’s success, our results show the main driver of success has been the CCSS’s ability to generate commitment to quality improvement through intrinsic motivators and interpersonal incentives. Intrinsic motivation has long been posited to be more effective in creating meaningful changes as compared to extrinsic (financial) motivators in the business and educational world (Deci *et al.*, 1999; Herzer and Pronovost, 2015). In part, intrinsic motivation in Costa Rica has been promoted by tapping into healthcare workers’ deep shared commitment and sense of accountability for achieving positive, equitable health outcomes (Pesec *et al.*, 2017b). The CCSS has positioned data collection and use as a key tool for achieving these outcomes, which has led to a culture that values data.

Intrinsic motivation in the Costa Rican health system is also consistent with the psychology of change work published by the Initiative for Healthcare Improvement, which posits that by activating an individual’s agency, one can create sustainable commitment to improvement (Hilton and Anderson, 2018). Costa Rica’s experience resonates with this framework, specifically Health Area’s autonomy to create their own quality improvement activities, distributing power to primary care providers and providing opportunities to co-design change. This autonomy, combined with professional pride and interpersonal incentives, unleashes providers’ intrinsic motivation for change and stimulates effective quality improvement.

Costa Rica’s experience in this regard is particularly valuable as RBF efforts have produced variable results, and countries around the world are looking to find alternate ways to generate buy-in without financial incentives (Paul *et al.*, 2018). RBF schemes have struggled to show lasting benefits and, in some cases, have exacerbated pre-existing disparities (Roberts *et al.*, 2017; Mendelson *et al.*, 2017; Lee *et al.*, 2012; Jha *et al.*, 2012). A common problem with RBF schemes is that non-incentivized conditions may be neglected (Campbell *et al.*, 2009). Costa Rica is not immune to this problem, and our informants did report that initiatives not assessed via the Index could be de-prioritized if measures in the Index needed to be improved. To guard against this, there are diverse PHC data streams in Costa Rica and various monitoring tools—such as the Local Management Plan—that track metrics not included in the Index. However, there is a constant tension between emphasizing various metrics, and it is the job of the Health Area Director to balance these competing demands in order to best serve its population-specific needs.

An additional enabler for data collection and use has been the CCSS’s focus on ensuring that all levels of healthcare professionals have robust education in management and data science. Around the world, the importance of training healthcare professionals in data collection and use has been shown. In Tamil Nadu, India, the Ministry of Health created an intensive training programme that includes an initial comprehensive training programme and targeted refresher trainings (Joint Learning Network for Universal Health Coverage, 2018). In Ghana, the Ministry of Health created a health informatics and biostatistics course for all healthcare professionals and used that opportunity to instil a culture of valuing data in its employees (Nakamura *et al.*, 2019; Joint Learning Network for Universal Health Coverage, 2018). Although management at the PHC level is a nascent field, recent studies corroborate our findings regarding the importance of management for effective PHC delivery. For example, one recent study on PHC facility management in Ghana showed that higher management scores were associated with better process and experiential outcomes (Macarayan *et al.*, 2019). The literature on how to improve management at the PHC level in low- and middle income countries is scarce (Mabuchi *et al.*, 2018). Costa Rica’s experiences in this arena may therefore be valuable to other countries seeking to strengthen PHC management and warrant further exploration.

While a culture of valuing data enables data-driven decision-making in Costa Rica, it has also led to the development of overlapping data streams that congest and hinder the system, acting as a barrier to effective data use and detracting time and resources from care. As Costa Rica integrates its new electronic medical record system with the existing data infrastructure, digitalization creates an opportunity for the system to simplify data systems and reduce the data collection burden. Other countries have had success with this strategy; after identifying duplication in their health information system, Bangladesh created a digitization plan that consolidates and harmonizes health information into one central technology platform DHIS2 (Bangladesh Ministry of Health and Family Welfare, 2017; Primary Health Care Performance Initiative, 2019; Garg and Garg, 2015). Furthermore, the possibilities of ‘big data’ to effectively organize and utilize the large amount of PHC data are abundant.

Limitations of this study include a limited sample size of informants and the time frame of the study, which did not allow us to seek saturation on every theme. We focused only on the data system as it existed during the time of data sampling and did not consider the impact of the new electronic medical record system implemented since 2017. Nonetheless, there is a great value in documenting and discussing the paper-based system as many countries still rely on paper medical records.

## Conclusion

As the global community recommits to PHC as the path to achieving Universal Health Coverage, measurement for improvement of PHC is critical. We describe Costa Rica’s overall data landscape, mapping the flow of nine different PHC data streams and how these data are used to drive population health management at the national, regional and local levels. Then, we describe in detail how one of those data streams, the Healthcare Delivery Performance Index, generates quality improvement. Strong training in data sciences and management support a culture that values data as a tool for improving population health, while complex and duplicative data systems act as a barrier to effective data use. We believe that Costa Rica’s experience in supporting data collection, analysis and use provides helpful insight for other countries looking to strengthen their measurement and improvement mechanisms.

## Abbreviations

ATAPs - Asistente Técnico de Atención Primaria, Community Health Workers

CCSS - Caja Costarricense de Seguro Social, the Costa Rican Social Security Administration

EBAIS - Equipos Básicos de Atención Integral de Salud, the Comprehensive Basic Primary Healthcare Teams

PHC - Primary Healthcare

RBF - Results-Based Financing

REDES - Registros de Salud Clerk, Medical Data Clerk

The Index - The Healthcare Delivery Performance Index

## Data Availability

The data underlying this article cannot be shared publicly for the privacy of individuals that participated in the study. The data will be shared on reasonable request to the corresponding author.
